# Correction for Siceloff et al., “Antimicrobial Resistance Hidden within Multiserovar *Salmonella* Populations”

**DOI:** 10.1128/aac.00926-25

**Published:** 2025-09-18

**Authors:** Amy T. Siceloff, Naomi Ohta, Keri N. Norman, Guy H. Loneragan, Bo Norby, H. Morgan Scott, Nikki W. Shariat

## AUTHOR CORRECTION

Volume 65, no. 6, e00048-21, 2021, https://doi.org/10.1128/aac.00048-21. Page 2, paragraph 3: “…(www.sankeymatic.com).” should read “…(www.sankeymatic.com). Figure 2A was generated using SankeyMatic (http://sankeymatic.com/build/). The Sankey plots were exported as high-resolution PNG files and the labels (cattle IDs and the percentage of serovar Reading on the left, and serovar name on the right) were added in PowerPoint. These files were saved as PDFs for submission.”

The authors would like to apologize for any confusion caused.

